# Management and outcome of metastatic pheochromocytomas/paragangliomas: a monocentric experience

**DOI:** 10.1007/s40618-021-01629-x

**Published:** 2021-07-05

**Authors:** G. De Filpo, G. Cantini, G. Rastrelli, G. Vannini, T. Ercolino, M. Luconi, M. Mannelli, M. Maggi, L. Canu

**Affiliations:** 1 Department of Experimental and Clinical Biomedical Sciences “Mario Serio”, University of Florence, Florence, Italy; 2 Endocrinology Unit, Careggi University Hospital, Florence, Italy

**Keywords:** PPGLs, Metastases, Overall survival, Wait and see, Treatment options

## Abstract

**Background:**

Pheochromocytoma (PHEO) and paraganglioma (PGL) are rare neuroendocrine tumors releasing catecholamines. Metastatic pheochromocytomas/paragangliomas (PPGLs) occur in about 5–26% of cases. To date, the management of patients affected by metastatic disease is a challenge in the absence of guidelines.

**Aim:**

The aim of this study was to evaluate the overall survival (OS) and the progression-free survival (PFS) in metastatic PPGLs.

**Methods:**

Clinical data of 20 patients referred to the Careggi University Hospital (Florence, Italy) were retrospectively collected. Follow-up ranged from 1989 to 2019. Site and size of primary tumor, biochemical activity, genetic analysis and employed therapies were considered. Data were analyzed with SPSS version 27.

**Results:**

Nine PHEOs (45%) and 11 PGLs (55%) were enrolled. Median age at diagnosis was 43.5 years [30–55]. Mean follow-up was 104.6 ± 89.3 months. Catecholamines were released in 70% of cases*.* An inherited disease was reported in 50% of patients. OS from the initial diagnosis (OSpt) and from the metastatic appearance (OSmtx) were lower in older patients (OSpt *p* = 0.028; OSmtx *p* < 0.001), abdominal PGLs (OSpt *p* = 0.007; OSmtx *p* = 0.041), larger tumors (OSpt *p* = 0.008; OSmtx *p* = 0.025) and sporadic disease (OSpt *p* = 0.013; OSmtx *p* = 0.008).

**Conclusion:**

Our data showed that older age at the initial diagnosis, sympathetic extra-adrenal localization, larger tumors and wild-type neoplasms are related to worse prognosis. Notably, the employed therapies do not seem to influence the survival of our patients. At present, effective treatments for metastatic PPGLs are missing and a multidisciplinary approach is indispensably required.

## Introduction

Pheochromocytomas and paragangliomas (PPGLs) are rare neural-crest derived tumors located in the adrenal medulla and extra-adrenal paraganglia, respectively. Among extra-adrenal tumors, sympathetic paragangliomas arise from the paraganglia of the thorax, abdomen and pelvis, while the parasympathetic paragangliomas originate from the head and neck paraganglia, named head and neck paragangliomas (HNPGLs). PPGLs’ incidence is about 0.6 cases per 100.000 persons/year and a germline or a somatic mutation in one of the susceptibility genes caused up to 50% of PPGLs [1].

The World Health Organization (WHO) classified PPGLs as malignant tumors because of their ability to metastasize [2, 3]. Approximately, 10% of pheochromocytomas (PHEOs) and 34% of paragangliomas (PGLs) are metastatic at diagnosis but distant spread can occur many years later [4, 5]. Metastatic PPGLs are characterized by high heterogeneity with a reported 5-year overall survival (OS) ranging from 40 to 77% [6, 7]. To date, different potential predictive factors of malignancy have been considered. A Ki67, nuclear antigen proliferative index, higher than 3% is most commonly found in metastatic disease, even if values lower than 3% have been reported [8]. The presence of germline mutations occurring in the *SDHB* gene, encoding the subunit B of the succinate dehydrogenase enzyme, was described in about 50% of patients affected by metastatic PPGLs and was related with disease progression and reduced survival [9, 10]. However, these results have not been confirmed in recent reports [11, 12]. Methoxytyramine (MTX), a metabolite of dopamine released by less differentiated tumors, has been proposed as a prognostic factor of metastatic spread [13]. Larger tumors, in particular greater than 50 mm, and extra-adrenal localization of the primary tumor were recognized as predictors of metastases development [14]. Nevertheless, the prognostic role of the PGLs site compared with PHEOs has not been confirmed in a large retrospective study on metastatic PPGLs [12].

In the absence of guidelines, the presence of catecholamines (CA)-related symptoms, the tumor burden and the disease progression have to be considered to choose the appropriate patient’s management. The “wait and see” approach may be employed in metastatic disease with indolent course and slow progression [4, 15, 16]. It has been demonstrated that surgical removal of the primary tumor can improve survival [11, 17] and reduce side effects due to the CA excess with decrease in cardiovascular comorbidities [15, 16]. Chemotherapy is usually employed in patients with high tumor burden and rapidly progressive disease. The scheme using cyclophosphamide–vincristine–dacarbazine (CVD) is the most utilized with no particularly encouraging results on improving survival [18, 19]. Temozolomide is effective in non-expressing O(6)-methylguanine-DNA methyltransferase (MGMT) tumors and *SDHB*-related PPGLs seem to be more responsive [20]. Sunitinib and other tyrosine kinase inhibitors (TKIs) may be utilized because of their ability to inhibit tumor angiogenesis and cell growth, but escape may occur after months or years following initial treatment through resistance development [21]. Radiation therapy can represent a good option to obtain local disease control [22]. Radiometabolic treatment using low specific activity (LSA) or high specific activity (HSA) ^131^I-MIBG or, alternatively, peptide receptor radionuclide therapy (PRRT) using ^90^Y or ^177^Lu-DOTATATE (Lutathera®), can be employed in patients with a positive ^123^I-MIBG or ^66^ Ga-DOTATOC scan, respectively [23, 24]. Long-acting somatostatin analogues (SSA), according to the high expression of somatostatin-receptor 2, should be assessed in the management of PPGLs [1]. In addition, various clinical trials are ongoing to evaluate the efficacy of targeted therapies.

The aim of this retrospective study was to appraise the prognostic factors of progression-free survival (PFS) and OS in a monocentric cohort of metastatic PPGLs.

## Materials and methods

### Population

Twenty patients affected by metastatic PPGLs were referred to the Endocrinology Unit of the Careggi University Hospital (Florence, Italy), between 1989 and 2019. They were enrolled and their clinical data have been retrospectively collected. Chromaffin disease was diagnosed by pathology after surgery of primary tumor (*n* = 19) or by instrumental and biochemical analyses when surgery was not performed (*n* = 1).

After patients gave their informed consent, genetic analysis was carried out. PFS was defined as the period from the surgery of primary tumor until the diagnosis of metastatic disease; OS was defined as the period from the initial diagnosis of chromaffin disease (OSpt) or from the appearance of metastases (OSmtx) to the last available follow-up (December 2019). PFS and OS differences according to localization and size of primary tumor, biochemical activity, genetic analysis and therapeutic choice employed (“wait and see” strategy vs intervention therapies) were taken into account.

### Statistical analysis

Data were analyzed with Statistical Package for Social Sciences (SPSS, Chicago, IL, USA), version 27. Continuous normally distributed variables were reported using mean and standard deviation (SD); continuous non-normally distributed variables were reported as median [interquartile range, IQR]. Categorical variables were expressed as percentages. PFS and OS were estimated using the Kaplan–Meier method. ROC curve analyses were used for evaluating tumor size accuracy as a prognostic factor. A *p* < 0.05 was considered statistically significant.

## Results

Twenty metastatic PPGLs (males/females, 45/55%) were included (Table 1). The median age at diagnosis of the primary tumor was 43.5 years [30–55], the mean follow-up was 104.6 ± 89.3 months. Nine (45%) PHEOs and 11 (55%) PGLs (6 abdominal PGLs, 5 HNPGLs) were observed. Four patients (20%) had multiple primary tumors at initial diagnosis. Median size of chromaffin lesions was 70 mm [31–80]. Catecholamines (CA) were released in 70% of cases; in particular, a predominantly noradrenergic secretion was found in 71.4% of tumors and a predominantly adrenergic secretion in 28.6% of cases [25]. A germline mutation in one of the susceptibility genes was detected in the half of patients: *SDHB* (15%), *SDHD* (10%), *SDHC* (10%), *PHD2* (5%), *MAX* (5%), and *KIF1Bβ* (5%).

**Table 1:** Demographic characteristics of patients

Characteristics	*N* evaluable	*N* total
Male/female (%)	20	9/11 (45/55)
Age at PHEO/PGL diagnosis—median [IQR]	20	43.5 [30–55]
Multiple primary tumors	20	4 (20)
Primary tumor localization (%)	20	
Adrenal		9 (45)
Extra-adrenal		11 (55)
Tumor secretion (%)	18	14 (70)
Adrenergic		4 (28.6)
Noradrenergic		10 (71.4)
Primary tumor size (mm)—median [IQR]	19	70 [31–80]
Genetic analysis (%)	20	
SDHB		3 (15)
SDHD		2 (10)
SDHC		2 (10)
PHD2		1 (5)
MAX		1 (5)
KIF1Bβ		1 (5)
Age at metastatic disease – median [IQR]	20	48.5 [35–64]
Synchronous metastases (%)	20	5 (25)
Metastatic site (%)	20	
Bone		12 (60)
Lymph nodes		8 (40)
Liver		6 (30)
Lungs		5 (25)
Local recurrence		4 (20)
Peritoneal dissemination		1 (5)
Treatments (%)	20	
Wait and see		5 (25)
Therapies		15 (75)
^131^I-MIBG		6 (30)
RT		6 (30)
PRRT		5 (25)
Surgery		5 (25)
Temozolomide		5 (33)
Sunitinib		4 (20)
CHT		3 (15)
SSA long-acting		2 (10)

*PHEO* pheochromocytoma, *PGL* paraganglioma, *CHT* chemotherapy, ^*131*^*I-MIBG* ^131^Iodine- metaiodobenzylguanidine, *RT* radiotherapy, *PRRT* peptide receptor radionuclide therapy, *SSA* somatostatin analogues

The median age at diagnosis of metastatic disease was 48.5 years [35–64]. Five patients (25%) had metastases at the initial diagnosis. Secondary spread involved bone (60%), lymph nodes (40%), liver (30%), lungs (25%), and peritoneum (5%). A locoregional relapse was observed in 20% of patients. Surgery for primary tumor was performed in 95% of cases. During follow-up, an approach with active surveillance (“wait and see”) was adopted in 25% of metastatic PPGLs, while in the remaining 75%, one or more intervention therapies were employed. Five patients (25%) underwent surgery to reduce the burden tumor, a ^131^I-MIBG radiometabolic treatment or a PRRT with ^177^Lu-DOTATATE was used in 30% (*n* = 6) and in 25% (*n* = 5) of cases, respectively. Six patients (30%) received radiotherapy. Furthermore, sunitinib was administered in 20% (*n* = 4), temozolomide (TMZ) in 33% (*n* = 5), long-acting SSA in 10% (*n* = 2) of cases. A chemotherapy was employed in 15% (*n* = 3) of cases, a regimen with etoposide plus cisplatin was used in one patient, while, in the remaining two patients, capecitabine was administered in one case and gemcitabine in the other.

Considering all patients, 5-year OSpt and OSmtx were 78.6% and 77.8%, respectively. OS at the last follow-up was 65%.

According to age tertiles (1st = 10–34 years; 2nd = 35–53 years; 3rd = 54–82 years), we found a significantly worse OSpt and OSmtx (p = 0.028 and p < 0.001, respectively) in the 3rd tertile.

**Fig. 1:**
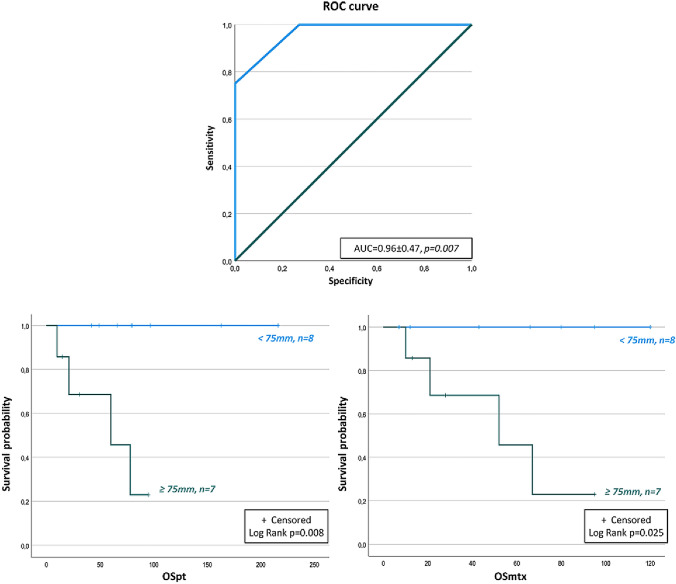
OS of patients with metastatic PPGLs according to tumor size threshold found through ROC curve analysis (75 mm). OSpt: overall survival from the diagnosis of primary tumor; OSmtx: overall survival from the diagnosis of metastatic disease

Considering that tumor diameter is a relevant prognostic factor, we tested in our population which was the best fitting tumor diameter in estimating OS by ROC curve analysis (Fig. 1 upper panel). Tumor diameter showed a very high accuracy (0.96 ± 0.47, *p* = 0.007) in estimating OS. In particular, for tumors ≥ 50 mm, the sensitivity in predicting death was of 100% and the specificity was of 63.6%. When a threshold of ≥ 75 mm in size was considered, the sensitivity was unchanged, but the specificity increased up to 72.7%. Figure 1 lower panels shows Kaplan–Meier analysis according to a tumor diameter ≥ 75 mm. Patients with this tumor size had lower estimated OS either from the diagnosis (OSpt *p* = 0.008) or from the first appearance of metastases (OSmtx *p* = 0.025). In contrast, Kaplan–Meier relationships of OS with tumor diameter ≥ 50 mm retained significance only for OSpt (*p* = 0.032) but not for OSmtx.

**Fig. 2:**
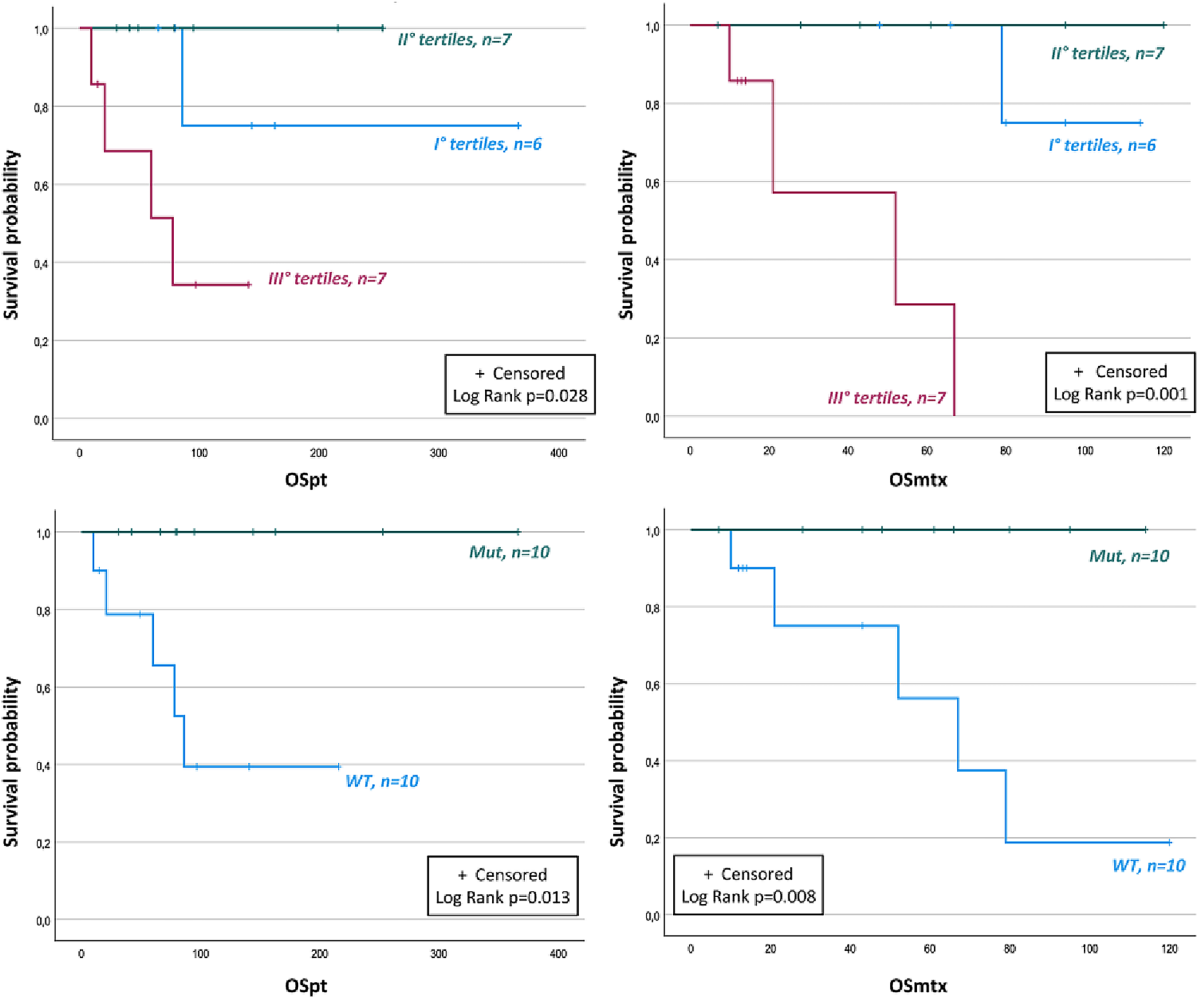
OS of patients with metastatic PPGLs according to age (tertiles) and genetic analysis. OSpt: overall survival from the diagnosis of primary tumor; OSmtx: overall survival from the diagnosis of metastatic disease; Mut: mutated; WT: wild type

Inherited diseases showed a significantly longer OSpt (*p* = 0.013) and OSmtx (*p* = 0.008) compared to sporadic ones. Germline carriers were younger than the wild-type counterpart (32.5 years [23.7–39.5] vs 55 years [52.2–68.2], *p* < 0.001). Wild-type group showed a OSpt at 5 years of 50% and of 37.5% at the last follow-up. Furthermore, OSmtx was 33.3% at 5 years and 16.7% at the end of the observation. No deaths were registered among mutation carriers. The levels of significance for the differences in OS according to age and genetic analysis are reported in Fig. 2.

No significant differences in PFS and OS were observed in mutated *SDHB* patients (PFS *p* = 0.263; OSpt *p* = 0.342; OSmtx *p* = 0.235) compared to other mutated patients, neither between secreting- and non-secreting tumors (PFS *p* = 0.200; OSpt *p* = 0.342; OSmtx *p* = 0.384).

**Fig. 3:**
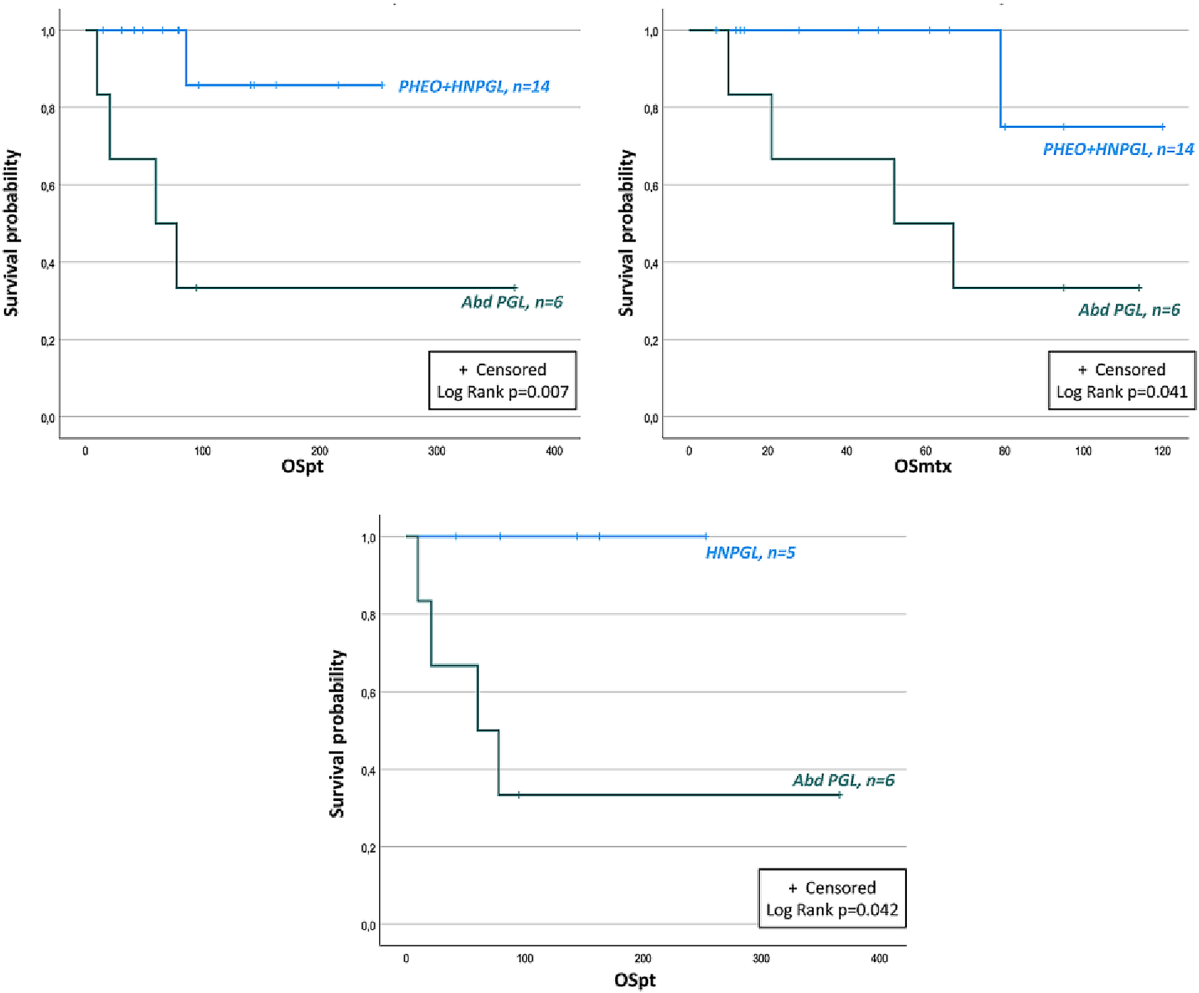
OS of patients with metastatic PPGLs according to the localization of primary tumor. PHEO: pheochromocytoma; HNPGL: head and neck paraganglioma; Abd PGL: abdominal paraganglioma; OSpt: overall survival from the diagnosis of primary tumor; OSmtx: overall survival from the diagnosis of metastatic disease

Patients affected by abdominal PGLs presented a lower OSpt and OSmtx compared to the others (PHEOs + HNPGLs) (*p* = 0.007 and *p* = 0.041, respectively). A lower OSpt was reported in abdominal PGLs compared to HNPGLs (*p* = 0.042). Abdominal PGLs had a median size significantly larger than all other evaluated tumors (85 mm [80–155] vs 33.5 mm [22.5–72.5], *p* = 0.003). Divergently, no differences in PFS and OS were reported in PHEOs vs PGLs (PFS *p* = 0.362; OSpt *p* = 0.286; OSmtx *p* = 0.277) and in PHEOs vs abdominal PGLs (PFS *p* = 0.806; OSpt *p* = 0.052; OSmtx *p* = 0.115). The levels of significance for the differences of OS according to the localization of the primary tumor are reported in Fig. 3.

Patients who underwent the “wait and see” strategy did not show lower OS compared to those in which different treatment options were employed (OSpt *p* = 0.276; OSmtx *p* = 0.227).

At the end of the study, a stable disease was observed in half of the patients (*n* = 9) who had a complete follow-up, while a progressive disease was reported in the other half (*n* = 9). During the entire observation period (366 months), five patients died (25%). In these patients, the median age at death was 71 years [40.5–79].

## Discussion

The management of metastatic PPGLs is a clinical challenge due to the lack of guidelines. Our retrospective study showed a lower OS from the initial diagnosis (OSpt) and from the metastatic diagnosis (OSmtx) in older patients, according to previous data [12]. Considering tumor localization, patients affected by abdominal PGLs experienced a lower OSpt and OSmtx compared to the group of patients affected by HNPGLs or PHEOs. In addition, a lower OSpt was reported in abdominal PGLs compared to HNPGLs. We noted that abdominal PGLs were significantly larger in size than other chromaffin lesions in our cohort of patients. Notably, no differences in PFS and OS were reported in PHEOs vs PGLs and in PHEOs vs abdominal PGLs.

The location of primary tumor should be carefully considered. In our patients, a worse prognosis in patients affected by abdominal PGLs has been shown when these tumors were compared to the group including both PHEOs and HNPGLs, but no significant prognostic differences were found between PHEOs vs PGLs and between abdominal PGLs vs PHEOs. As expected, parasympathetic lesions showed an indolent course and improved prognosis compared with abdominal PGLs [26, 27]. HNPGLs are generally not biochemically active and determine the compression of surrounding structures without side effects due to CA excess [28]. These favorable characteristics may represent an important survival-influencing factor. In a large retrospective multicentric study on metastatic PPGLs promoted by ENS@T group (MAPP Prono study) [12], the prognostic role of PGLs vs PHEOs site in metastatic PPGLs was not confirmed, suggesting that extra-adrenal location include tumors with different prognostic behaviors. For this reason, HNPLGs might represent as a new favorable prognostic category.

Furthermore, we found that primary tumor size and genetic analysis influenced the prognosis. In particular, tumors ≥ 50 mm were related with lower OSpt. A lower OSpt and OSmtx were reported in patients with tumors ≥ 75 mm, a threshold obtained through ROC curve analysis. Our data confirmed the prognostic value of the primary tumor size as previously reported [11, 14, 17]. It could be useful to perform a ROC curve to identify a specific prognostic size threshold in each cohort of patients.

In line with the MAPP Prono study [12], our data showed a worse prognosis in wild-type patients, in which a lower OSpt and OSmtx was reported. Specifically, in our series germline carriers were younger than the wild-type counterpart (median age: 32.5 [23.7–39.5] vs 55 [52.2–68.2] years, *p* < 0.001), this evidence might influence survival results. Intriguingly, *SDHB* germline mutation did not worsen the prognosis. Mutations occurring in the *SDHB* gene are more frequently associated with metastatic spread [13, 29], but recent evidences showed that the presence of *SDHB* mutations did not correlate with the prognosis in patient with a metastatic disease [11, 12]. Among our patients, only three were *SDHB* carriers, this small number could represent a limit to evaluate the prognostic impact of *SDHB* mutations. In particular, the median age at diagnosis was 30 years [10–37] and two out of three *SDHB* patients presented non-secreting lesions (HNPGLs). These favorable prognostic factors might justify our results.

No prognostic divergences were found between non-secreting and secreting lesions. Moreover, no differences were detected also between adrenergic and noradrenergic tumors. It is known that noradrenergic-secreting tumors do not express phenylethanolamine-N-methyltransferase (PNMT) enzyme; therefore, they are less differentiated than adrenergic-secreting lesions [30, 31]. It has been reported that patients with metastatic disease have levels five times higher of methoxytyramine (MTX), a dopamine metabolite produced by undifferentiated tumors, compared to patients with indolent disease [13]. Unfortunately, we did not have complete data about MTX values in our population.

In the literature, controversial data were reported about the prognostic relevance of Ki67, nuclear antigen proliferative index. A value greater than 3% is associated with an increased risk of metastases but cases of metastatic disease with Ki67 < 3% have been described [8]. In fact, many PPGLs have a low proliferative index even if capable of metastatic spread [31]. In our series, Ki67 percentage was available in only a few cases; therefore, it was not possible to analyze how this factor influenced the prognosis in our population.

Finally, we evaluated OS comparing the group of patients who had undergone therapies for metastatic disease and the group of patients in which a biochemical and instrumental follow-up was chosen. We did not find significant differences in OSpt and OSmtx between these two cohorts. This is a very interesting result, but it is important to highlight the numerical gap between these two groups (15 vs 5 patients, respectively). Multicentric studies including larger cohorts of patients are needed to deeply assess the relevance of this aspect, also through appropriate statistical analyses.

Our study has several limitations. First, the retrospective design did not permit us to obtain complete information about Ki67 and MTX evaluation. Second, the small number of the enrolled patients represents another limitation, which may prevent to unveil possible predictors and to perform Cox analyses to identify independent predictors of PFS and OS, although the rarity of chromaffin tumors should be considered.

## Conclusion

Our data showed that an older age at diagnosis, a larger size of the primary tumor and a presence of a sporadic disease represent negative prognostic factors. The extra-adrenal localization of primary tumor might be carefully considered and the prognostic relevance of *SDHB* mutations might be reassessed. Furthermore, a “wait and see” approach might be contemplated in patients with low tumor burden and slowly progressive disease. We recommend choosing interventional therapies case-by-case regarding side effects due to catecholamines excess and associated comorbidities. The management of metastatic PPGLs is a great clinical challenge; therefore, a multidisciplinary approach is indispensably required.

## Abbreviations

PHEO: Pheochromocytoma
PGL: Paraganglioma
HNPGLs: Head and neck paragangliomas
PPGLs: Pheochromocytomas/paragangliomas
MTX: Methoxytyramine
OSpt: Overall survival from the diagnosis of primary tumor
OSmtx: Overall survival from the diagnosis of metastases
PFS: Progression free survival
^131^I-MIBG: ^131^Iodine-metaiodobenzylguanidine
^90^Y or ^177^Lu-DOTATATE: ^90^Yttrium or ^177^Lutetium-DOTA0-Tyr^3^-octreotate
^66^Ga-DOTATOC: ^66^Gallium-DOTA0-Tyr^3^-octreotide
PRRT: Peptide receptor radionuclide therapy
SSA: Somatostatin analogues
